# Clade-Specific Sterol Metabolites in Dinoflagellate Endosymbionts Are Associated with Coral Bleaching in Response to Environmental Cues

**DOI:** 10.1128/mSystems.00765-20

**Published:** 2020-09-29

**Authors:** Yandu Lu, Jiaoyun Jiang, Hongwei Zhao, Xiao Han, Yun Xiang, Wenxu Zhou

**Affiliations:** a State Key Laboratory of Marine Resource Utilization in the South China Sea, College of Oceanology, Hainan University, Haikou, Hainan, China; b Shandong Rongchen Pharmaceuticals Inc., Qingdao, China; c College of Life Sciences, Guangxi Normal University, Guilin, Guangxi, China; Vanderbilt University

**Keywords:** coral bleaching, symbiosis, sterols, *Heteractis crispa*, Symbiodiniaceae

## Abstract

Cnidarians cannot synthesize sterols (which play essential roles in growth and development) *de novo* but often use sterols acquired from endosymbiotic dinoflagellates. While sterol availability can impact the mutualistic interaction between coral host and algal symbiont, the biosynthetic pathways (in the dinoflagellate endosymbionts) and functional roles of sterols in these symbioses are poorly understood. In this study, we found that itraconazole, which perturbs sterol metabolism by inhibiting the sterol 14-demethylase CYP51 in dinoflagellates, induces bleaching of the anemone Heteractis crispa and that bleaching perturbs sterol metabolism of the dinoflagellate. While Symbiodiniaceae have clade-specific sterol metabolites, they share features of the common sterol biosynthetic pathway but with distinct architecture and substrate specificity features of participating enzymes. Tracking sterol profiles and transcripts of enzymes involved in sterol biosynthesis across time in response to different environmental cues revealed similarities and idiosyncratic features of sterol synthesis in the endosymbiont Breviolum minutum. Exposure of algal cultures to high levels of light, heat, and acidification led to alterations in sterol synthesis, including blocks through downregulation of squalene synthase transcript levels accompanied by marked growth reductions.

**IMPORTANCE** These results indicate that sterol metabolites in Symbiodiniaceae are clade specific, that their biosynthetic pathways share architectural and substrate specificity features with those of animals and plants, and that environmental stress-specific perturbation of sterol biosynthesis in dinoflagellates can impair a key mutualistic partnership for healthy reefs.

## Abstract (precis)

These results indicate that sterol metabolites in Symbiodiniaceae are clade specific, that their biosynthetic pathways share architectural and substrate specificity features with those of animals and plants, and that environmental stress-specific perturbation of sterol biosynthesis in dinoflagellates can impair a key mutualistic partnership for healthy reefs.

## INTRODUCTION

Corals and sea anemones are marine invertebrates of the phylum Cnidaria. Many are symbiotic species that engage as hosts in mutualistic partnerships (symbioses) with photosynthetic, algal endosymbionts (1). The major symbiotic algae associated with cnidarians are members of the dinoflagellate family Symbiodiniaceae, which has high phylogenetic diversity (2). Annotated genomes are available for several taxa of the family, including Symbiodinium microadriaticum (clade A) (3), Breviolum minutum (clade B, formerly S. minutum) (4), Cladocopium goreaui (clade C, formerly S. goreaui) (5), and Fugacium kawagutii (clade F, formerly S. kawagutii) (6). The dinoflagellate endosymbionts supply their cnidarian hosts with photosynthetic products (7) while benefiting from using CO_2_ and nutrients in the host’s waste products and their stable position in the water column with exposure to light (1). As the reefs provide numerous socioeconomic and ecological benefits in addition to high biodiversity (*inter alia*, tourist and recreational attractions, shoreline protection, and fisheries), there are urgent needs for detailed investigations of the establishment and functioning of the mutualistic partnerships between dinoflagellate endosymbionts and cnidaria.

Sterols are vital components of all eukaryotic cells (8). They play key roles in cell viability, embryogenesis, pattern formation, cell division, chloroplast biogenesis, and modulation of the activity and distribution of membrane-bound proteins, such as enzymes and receptors (9). In addition, sterols are precursors of many signaling molecules that regulate growth and development in plants and animals, such as insect ecdysteroid molting hormones (10), mammalian steroid hormones (11), and plant brassinosteroid hormones (12). Thus, all animals need sterols, particularly cholesterol, to survive. Plants produce phytosterols, which can be converted by sterol-auxotrophic invertebrates (e.g., insects and nematodes) to cholesterol by removal of the ethyl or methyl group on the sterol side chain (13). In contrast, C4-methylated sterols cannot be converted to cholesterol or utilized by these invertebrates (14–17).

While it is understood that symbiotic cnidarians cannot synthesize sterols *de novo* (18), diverse sterols have been identified in both cnidarians (19, 20) and the dinoflagellates residing within the host cells of many cnidarian species (21). Symbiotic cnidarians appear to acquire sterols from their symbionts. Homologs of sterol-trafficking Niemann-Pick type C (NPC) proteins involved in sterol transport have been identified via *in silico* analyses in anemones (22). The noncanonical NPC2 proteins have recently demonstrated symbiosis specificity and gradually accumulate in the symbiosome during evolution under selective pressure to mediate sterol transfer between hosts and symbionts (23). These observations suggest that sterols synthesized by the dinoflagellate endosymbionts are transported into the host cytosol (22, 24, 25). This inference, together with other lipidomic, biochemical, and pharmacological observations (19, 23, 26), supports the hypothesis that sterols in the coral polyps are biosynthetic products processed by the assimilation or modification of dietary metabolites, or metabolites produced by endosymbionts, and that these exchanges are crucial for stable symbiosis.

The sea anemone Heteractis crispa is the most highly traded species of sea anemone worldwide due to its beautiful colors, which range from gray or purple to violet-brown, depending on the concentration of its symbiotic dinoflagellate (27). It is widespread throughout the tropical and subtropical waters of the Indo-Pacific region from the eastern coasts of Africa to Polynesia and from southern Japan to Australia and New Caledonia (28). It provides habitat for 14 of the 26 species of clownfish (29). Therefore, H. crispa has high economic and ecological values (27, 29). Moreover, H. crispa requires low levels of feeding for maximal growth, which is also highly dependent on the nutrients provided by dinoflagellate endosymbionts (30). This characteristic is advantageous for studying the metabolic interaction between cnidarian hosts and their symbionts in this system.

In this study, we explored the roles of sterol metabolites in the mutualistic partnerships between cnidarian hosts and dinoflagellate endosymbionts, by probing effects of perturbing sterol metabolism in the symbionts of H. crispa. Using the dinoflagellate endosymbiont B. minutum, we also investigated sterol biosynthesis pathways and their roles in responses to bleaching-inducing stresses. Our findings improve the understanding of the dynamics of sterol biosynthesis and metabolism in Symbiodiniaceae and their role in maintaining the integrity of their symbioses with cnidarians.

## RESULTS

### Cnidarian hosts cannot synthesize sterols *de novo* but have complex sterol profiles.

Many marine invertebrates, including members of the phylum Cnidaria, are known to lack the ability to synthesize sterols *de novo*, despite requiring them like all eukaryotes (31). To acquire more information about this deficiency, and about the mechanisms whereby these invertebrates acquire sterols, we examined suites of isoprenoid and sterol biosynthesis genes in genomes of six selected organisms. These were two cnidarians—a stony coral of the Acroporidae family, Acropora digitifera (32), and a sea anemone of the Aiptasiidae family, *Exaiptasia* sp. (22)—and the following four endosymbionts of cnidarians, each representing a different clade: B. minutum, C. goreaui, F. kawagutii, and Symbiodinium microadriaticum (see Fig. S1a in the supplemental material) (2). In the endosymbionts, a complete core sterol biosynthetic pathway was detected, while in cnidarians, no copies were found of genes that are generally essential for sterol biosynthesis. For example, no genes encoding sterol 14-demethylase (CYP51) (a key enzyme in sterol biosynthesis [33]) and squalene synthase (SQS) (which catalyzes the first committed step in sterol formation [34]) were found in the selected cnidarians (Fig. S1b; see also Data Set S1 in the supplemental material). In line with previous studies (19, 22–26, 35), our findings support the idea of the presence of a core sterol biosynthetic pathway in the endosymbionts and its absence in cnidarians (including both sterol C4-methylation and C4-demethylation capacities).

10.1128/mSystems.00765-20.1 FIG S1 Conservation of sterol biosynthetic genes in cnidarian hosts and Symbiodiniaceae. (a) Phylogenetic relationships between major clades of Symbiodiniaceae. Red symbols indicate clades with strains used in this study. (b) Comparison of sterol biosynthesis results in cnidarian hosts and Symbiodiniaceae. The color key (top right) indicates the similarity of the gene to the closest match in *Arabidopsis* and ranges from low (white) to high (red) similarity. White areas indicate no Blastp hit below the applied E value threshold (1e−5). Red areas indicate orthologs with Blastp E values below 1e−100. Color in the heat map is scaled columnwise based on the bit values of tblastn results (Data Set S1). See Materials and Methods for details. Enzyme abbreviations: HMGS, hydroxy-methylglutaryl-CoA (hydroxy-methylglutaryl-coenzyme A) synthase; HMGR, hydroxy-methyl-glutaryl-CoA reductase; MVK, mevalonate kinase; PMK, phosphomevalonate kinase; DXS, 1-deoxy-d-xylulose 5-phosphate synthase; DXR, 1-deoxy-d-xylulose 5-phosphate reductoisomerase; CMS, 4-diphosphocytidyl-2C-methyl-d-erythritol synthase; CMK, 4-(cytidine 5′-diphospho)-2-C-methyl-d-erythritol kinase; MDS, 2C-methyl-d-erythritol 2,4-cyclodiphosphate synthase; HDS, hydroxy-2-methyl-2-(E)-butenyl 4-diphosphate synthase; HDR, hydroxy-2-methyl-2-(E)-butenyl 4-diphosphate synthase; IPI, isopentenyl diphosphate:dimethylallyl diphosphate isomerase; GPS, geranyl diphosphate synthase; FPS, farnesyl diphosphate synthase; GGPPS, geranylgeranyl diphosphate synthase; SQS, squalene synthase; LAS, lanosterol synthase; STRM, sterol A-ring methylase-1; SMT, sterol methytransferase; CPI, cycloeucalenol cycloisomerase; CYP51, sterol 14-alpha demethylase; FK, sterol C-14 reductase; DWF7, delta7 sterol C-5 desaturase; DWF5, sterol C-7 reductase; DWF1, sterol C-24(28) isomerase-reductase. Abbreviations for species: Ad, Acropora digitifera; Bm, Breviolum minutum; Cg, Cladocopium goreaui; Ex, *Exaiptasia* sp.; Fk, Fugacium kawagutii; Sm, Symbiodinium microadriaticum. Note that the light red corresponding to some homologues (e.g., DWF7 in Symbiodiniaceae) indicates the low but significant similarity in studied species to corresponding *Arabidopsis* enzymes and that the question marks indicate that data representing the presence or absence of corresponding enzymes require biochemical validation. Download FIG S1, PDF file, 0.1 MB. Copyright © 2020 Lu et al. 2020 Lu et al. This content is distributed under the terms of the Creative Commons Attribution 4.0 International license.

10.1128/mSystems.00765-20.8 DATA SET S1 List of proteins involved in the sterol biosynthetic pathway used to create the similarity heat map shown in Fig. S1. A local blast database was constructed, and inferred proteins from all analyzed genomes were subjected to blast analyses against *Arabidopsis* or human proteins. Blast results were parsed for ≥25% amino acid identity with E values of ≤1e−10. Sequences whose real identity could not be confirmed were removed manually. Abbreviations for species: Ad, *Acropora digitifera*; Bm, *Breviolum minutum*; Cg, *Cladocopium goreaui*; Ex, *Exaiptasia* sp.; Fk, *Fugacium kawagutii*; Sm, *Symbiodinium microadriaticum*. Download Data Set S1, XLSX file, 0.01 MB. Copyright © 2020 Lu et al. 2020 Lu et al. This content is distributed under the terms of the Creative Commons Attribution 4.0 International license.

Sterol profile analysis revealed 18 sterols in H. crispa, designated H1 to H18, after removal of the symbiotic algae (here referred to as alga-freed H. crispa) (see Table S1 in the supplemental material; see also Fig. S2a). The most abundant was cholesterol (∼1.3 μg·mg^−1^ dry weight [DW]; H2) (see Fig. S2a for molecular structure). Other sterols were mostly side chain- and/or nucleus-alkylated sterols, likely originating from the dinoflagellates. We detected abundant *Cladocopium* spp. in the H. crispa anemones (18S ribosomal DNA [rDNA] accession number MN934820), but attempts to culture this alga directly isolated from these anemones were unsuccessful. Therefore, for comparison we analyzed sterols in H. crispa with the symbiotic algae (whole organism) (Table S1). The overall sterol profile was very similar to that of the alga-freed animals, including the same set of 18 sterols (Table S1). These findings provide little indication of the extent to which algal sterols were transferred between species, but we observed higher levels of total sterols in the alga-freed organisms (19.3 ± 0.03 μg·mg^−1^ DW) than in the whole organisms (15.9 ± 0.6 μg·mg^−1^ DW) (Table S1). The difference in sterol levels could be due to the presence or absence of algae in the anemone. This could have been due to the algae containing less sterol than the host organism per unit mass. The whole animals contained slightly less cholesterol (1.022 ± 0.02 μg·mg^−1^ DW) than the alga-freed animals (1.287 ± 0.004 μg·mg^−1^ DW) (Table S1). This suggests that cholesterol is crucial for biological processes in the anemone, as in other animals (13).

10.1128/mSystems.00765-20.2 FIG S2 Identification of sterol compounds extracted from H. crispa, B. minutum, and F. kawagutii. (a) Structures and mass spectra of sterols identified from H. crispa. (b) Structures and mass spectra of sterols identified from F. kawagutii. (c) Structures and mass spectra of sterols identified from B. minutum. (d) Structures and mass spectra of sterols identified from B. minutum treated with sterol biosynthesis inhibitors. Download FIG S2, PDF file, 0.6 MB. Copyright © 2020 Lu et al. 2020 Lu et al. This content is distributed under the terms of the Creative Commons Attribution 4.0 International license.

10.1128/mSystems.00765-20.6 TABLE S1 Sterol profiles of whole or alga-freed (i.e., with symbiotic algae removed) Heteractis crispa. Three biological replicates of healthy anemones were cultured in seawater with pH 8.2 at 24 to 26°C and 50 μmol·photons·m^−2^·s^−1^ light. They were fed with brine shrimp semimonthly and then starved approximately 10 days before the experiments to avoid sample contamination by food metabolites. Portions of cultures were collected, while zooxanthellae were removed by a scraping technique (Alga-freed) or were left in the tissue (Whole) for sterol profiling performed with three biological replicates (see Materials and Methods for details). Average values ± errors are shown. Download Table S1, DOCX file, 0.02 MB. Copyright © 2020 Lu et al. 2020 Lu et al. This content is distributed under the terms of the Creative Commons Attribution 4.0 International license.

### Impairment of sterol biosynthesis in endosymbionts induces cnidarian bleaching and vice versa.

**FIG 1:**
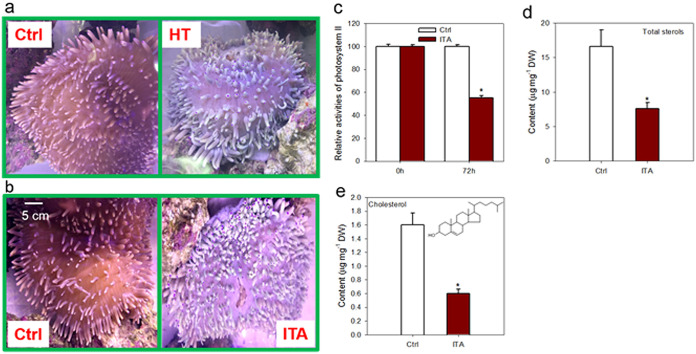
Process of bleaching of the cnidarian H. crispa induced by sterol starvation. Healthy cultures were transferred into fresh media and acclimated for at least 2 months before experimentations. Specimens were fed with brine shrimp semimonthly. They were starved approximately 10 days before the experiments to avoid sample contamination by food metabolites. Control anemones that were not subjected to the stresses were processed in a manner identical to that used for the test samples. See details in Materials and Methods. (a) Comparison of healthy and high-temperature (HT)-stressed H. crispa anemones. Healthy H. crispa anemones were cultured in a tank in 26°C (control [Ctrl]) or 34°C (HT) for 72 h. (b) Comparison of healthy (Ctrl) and sterol-starved (ITA) H. crispa anemones. Healthy H. crispa anemones cultured in a tank received the inhibitor itraconazole (ITA, 5 mg · liter^−1^) or an equivalent amount of DMSO (not exceeding a final concentration of 0.1%; Ctrl) for 72 h. (c) Relative levels of maximum photosynthetic efficiency of photosystem II (PSII) of H. crispa algal endosymbionts in response to sterol starvation induced by ITA feeding. (d) Levels of total sterols in healthy and ITA-fed H. crispa. (e) Changes in cholesterol contents of H. crispa following ITA feeding. Asterisks (*) indicate significant differences compared with the control conditions (*P* ≤ 0.05).

To examine effects of bleaching on sterol homeostasis, we next analyzed sterol profiles of bleached H. crispa where the bleaching resulted from high temperature (Fig. 1a). The bleached anemones had the same types of sterols as unbleached controls but had substantially lower quantities of total sterols and cholesterol (24.6% and 41.6% reductions, respectively; Table 1). Thus, sterol metabolism in H. crispa is altered by bleaching induced by high temperature. Since itraconazole (ITA) is a highly specific inhibitor of CYP51 (36) and since no *CYP51* gene was detected in the screened cnidarian genomes (Fig. S1b), it should theoretically disrupt algal sterol production only in H. crispa hosting algal endosymbionts. Pharmaceutical perturbation of sterol homeostasis by ITA led to bleaching of the sea anemone H. crispa (Fig. 1b) and a dramatic reduction in the photosynthetic capacity of cultured algal (B. minutum) endosymbionts (Fig. 1c). Similarly to the pattern observed in the high-temperature-bleached anemones, ITA treatments led to a 54% decrease in total sterol content (Table 1; see also Fig. 1d) and a 76% reduction in cholesterol levels, from 1.60 ± 0.17 to 0.60 ± 0.06 μg·mg^−1^ DW (Fig. 1e), in the alga-freed H. crispa. In addition, there were significant reductions in brassicasterol (sterol H4) levels and increases in the levels of 24-methylcholesterol (H8) and 24-methylcholesta-5,7,24-trienol (H10) in response to both high-temperature and bleaching processes (Table 1). Thus, perturbation of sterol biosynthesis in H. crispa endosymbionts induces cnidarian bleaching and vice versa.

**TABLE 1:** Sterol profiles of the sea anemone H. crispa^*a*^ under heat stress conditions or treated with itraconazole (ITA)^*b*^

Sterol	Structure^*d*^	Content (% of sterols)			
		Ctrl^*c*^	Heat	Ctrl^*c*^	ITA
24-Nor-23-demethylgorgosterol	**H1 (B1)**	0.22 ± 0.03	0.12 ± 0.01	0.14 ± 0.02	0.14 ± 0.01
Cholesterol	**H2 (B2)**	9.63 ± 0.57	7.46 ± 0.56*^,^ ^*e*^	9.63 ± 0.07	7.86 ± 1.02*
Cholestanol	**H3**	0.17 ± 0.02	0.11 ± 0	0.2 ± 0.03	0.14 ± 0.03
Brassicasterol	**H4 (B4)**	2.35 ± 0.01	1.86 ± 0.08*	1.96 ± 0.06	1.68 ± 0.06*
24-Methylcholesta-7,9,22-trienol	**H5**	1.46 ± 0.11	0.94 ± 0.07*	1.52 ± 0.14	0.68 ± 0.07*
24-Methylcholesta-5,7,22-trienol	**H6**	0.79 ± 0.04	0.64 ± 0.05*	0.31 ± 0.04	0.27 ± 0.11
24-Methylenecholesterol	**H7**	71 ± 0.34	68.3 ± 0.44	73.5 ± 0.05	74.1 ± 0.93
24-Methylcholesterol	**H8**	6.1 ± 0.01	7.52 ± 0.32*	7.13 ± 0.05	9.15 ± 0.05*
24-Ethylcholesta-5,22-dienol	**H9**	0.26 ± 0.02	0.36 ± 0.02*	0.23 ± 0	0.21 ± 0
24-Methylcholesta-5,7,24-trienol	**H10**	3.56 ± 1.05	7.19 ± 1.44*	1.7 ± 0.47	2.3 ± 1.77*
24-Methylcholesta-5,7-dienol	**H11**	1.71 ± 0.16	2.56 ± 0.25*	0.94 ± 0.15	0.96 ± 0.46
24-Methylcholsta-7,24(28)-dienol	**H12**	0.1 ± 0.01	0.13 ± 0.02	0.1 ± 0.03	0.15 ± 0
24-Ethylcholest-7,22-dienol	**H13**	1.05 ± 0	1.48 ± 0.15	1.14 ± 0.29	1.07 ± 0.13
24-Ethylcholest-5-enol	**H14**	0.22 ± 0.05	0.18 ± 0.04	0.2 ± 0	0.17 ± 0.02
4,24-Dimthylcholest-7-enol	**H15 (B10)**	0.27 ± 0.01	0.35 ± 0.02	0.26 ± 0.03	0.3 ± 0.05
4,24-Dimethycholestanol	**H16 (B12, F6)**	0.05 ± 0	0.07 ± 0.01	0.08 ± 0.03	0.1 ± 0.02
Dinosterol	**H17 (B13, F7)**	0.02 ± 0.01	0.07 ± 0.01	0.07 ± 0.01	0.02 ± 0.01
4,23,24-Trimethylcholest-7-enol	**H18**	0.02 ± 0	0.04 ± 0	0.03 ± 0	0.05 ± 0.01

Total sterols (μg·mg^-1^ DW)		14.2 ± 0.2	10.7 ± 0.6*	16.6 ± 2.4	7.6 ± 0.9*

a Anemones were fed with brine shrimp semimonthly and then starved for approximately 10 days before the experiments to avoid sample contamination by food metabolites. Specimens that were not subjected to the stresses served as controls. These controls were processed in a manner identical to that used for the tested samples to maximally eliminate potential food contamination. Asterisks (*) indicate significant differences compared with the control conditions (Ctrl; *P* ≤ 0.05).

b The heat or ITA treatments were applied as described in Materials and Methods. For the heat treatments, sea anemones were transferred from preferred conditions to seawater with pH 8.2 at 34°C and 50 μmol·photons·m^−2^·s^−1^ light. As for the ITA treatments, cultures were treated with inhibitors (5 mg · liter^−1^) or an equivalent amount of DMSO (controls; not exceeding a final concentration of 0.1%). Portions of cultures were collected, while zooxanthella cells were removed by the use of a scraping technique for sterol profiling at the indicated times.

c The heat and ITA treatments were not conducted at the same time, which led to variations between the controls.

d The structure numbers are indicated in boldface, and their corresponding mass spectra are listed in Fig. S2a. “H,” “B,” and “F” indicate sterols identified in the anemone H. crispa, B. minutum, and *F. kawagutii*, respectively. For molecules identified in two or more species, the symbols for the molecules in other biological samples are shown in parentheses.

e Three biological replicates were established under each set of treatment conditions. Average values ± errors are shown.

### Clade-specific sterol metabolites in Symbiodiniaceae.

The preceding evidence indicates that sterol metabolism in the endosymbiont substantially affects (and may be essential for the integrity of) H. crispa hosting it. Thus, understanding the sterol biosynthesis pathway in dinoflagellate endosymbionts is crucial for thorough understanding of their relationship and of the processes that affect the organisms both individually and symbiotically. Therefore, we surveyed the sterol composition of two endosymbiotic dinoflagellate species representing different genera, B. minutum (clade B; isolated from the Caribbean coral Montastraea faveolata in Tennessee Reef, FL, USA) (4) and F. kawagutii (clade F; isolated from the North Pacific scleriactinian coral Montipora verrucosa) (6). Seventeen sterols were detected, with some overlap in the sets, including 14 in B. minutum (designated B1 to B14) and 7 in F. kawagutii (designated F1 to F7) (Table 2). These included a mixture of C4-methyl sterols and C4-desmethyl sterols, with differences in the dominant sterols and biosynthetic intermediates (listed in Table 2, labeled in bold in the following text, and with structures shown in Fig. S2). A few sterols (B5, B8, B12, and B13) were detected in both species, suggesting they may participate in essential biological processes in Symbiodiniaceae. In *F. kawagutii*, dinosterol (F7; see Fig. S2b for molecular structure) was the most abundant, comprising ∼40.4% of the total amount (Table 2). Interestingly, all sterols isolated from *F. kawagutii* were C4-methylated forms, which cannot be converted by the cnidarian host to cholesterol, as described above in the introduction.

**TABLE 2:** Comparison of sterol profiles between *B. minutum* and *F. kawagutii*^*a*^

Sterol	Structure	*B. minutum* (%)	*F. kawagutii* (%)
24-Nor-23-demethylgorgosterol	**B1 (H1)** ^*b*^ ^,^ ^*c*^	0.9	—^*d*^
Cholesterol	**B2 (H2)** ^*c*^	10.6	—
23,24-Demethylgorgosterol	**B3** ^*c*^	0.4	—
Brassicasterol	**B4 (H4)** ^*c*^	2.8	—
4-Methylcholest-22-enol	**F1**	—	6.9
Lophenol	**B5 (F2)**	0.5	7.5
Lophanol	**F3**	—	5.3
22-Dihydrobrassicasterol	**B6**	5.4	—
4,23-Dimethylcholest-7-enol	**F4**	—	13.3
24-Demethylgorgosterol	**B7** ^*c*^	1.9	—
24-Demethyldinosterol	**B8 (F5)**	27.2	18.4
4,24-Dimethylcholesta-7,22-dienol	**B9**	0.5	—
4,24-Dimethylcholest-7-enol	**B10 (H15)**	0.9	—
23,24-Demethylcholesta-5,22-dienol	**B11** ^*c*^	0.6	—
4,24-Dimenthylcholestanol	**B12 (F6, H16)**	18.8	8.3
Dinosterol	**B13 (F7, H17)**	12.4	40.4
Gorgosterol	**B14** ^*c*^	17.0	—

a Cultures in exponential phase (1 × 10^6^ cells·ml^−1^) were collected for sterol profiling. Three biological replicates of algal cultures were established.

b The structure numbers in boldface and their corresponding mass spectra are listed in Fig. S2b and c. “H,” “B,” and “F” indicate sterols identified in the anemone H. crispa, B. minutum, and *F. kawagutii*, respectively. For molecules identified in two or more species, their designations in other biological samples are shown in parentheses.

c Δ^5^ sterol.

d Dashes (—) indicate values below the detection limit.

The sterol profiles in B. minutum were more complex, and 24-demethyldinosterol (B8; see Fig. S2c for molecular structure) was the most abundant of its 14 detected sterols, accounting for 27.2% of the total content (Table 2). A large proportion of B. minutum sterols were Δ^5^ sterol variants, including cholesterol (B2), which can be directly used by the host (Table 2). Despite their close phylogenetic relationship, we found substantial differences in the dominant sterol types and biosynthetic intermediates between B. minutum and *F. kawagutii* (Table 2). Although cholesterol accounted for a substantial proportion of the total sterols in H. crispa (∼9.63%; Table 1) and B. minutum (∼10.6%; Table 2), it was absent in *F. kawagutii* (Table 2). In addition, all sterols isolated from *F. kawagutii* were C4-methylated species (which cannot be converted by the cnidarian to cholesterol). Thus, sterol profiles do not match the metabolic capabilities of a cnidarian host and *F. kawagutii* may be incapable of colonizing and establishing a stable symbiosis with cnidarian hosts. This hypothesis is also supported by recent genomic evidence (2, 5). Thus, we selected B. minutum to further study the architecture of the sterol biosynthetic pathway and roles of sterols in the response of dinoflagellate endosymbionts to environmental stresses that induce coral bleaching.

### The Symbiodiniaceae sterol biosynthetic pathway shares architectural and substrate specificity features with those of animals and plants.

**FIG 2:**
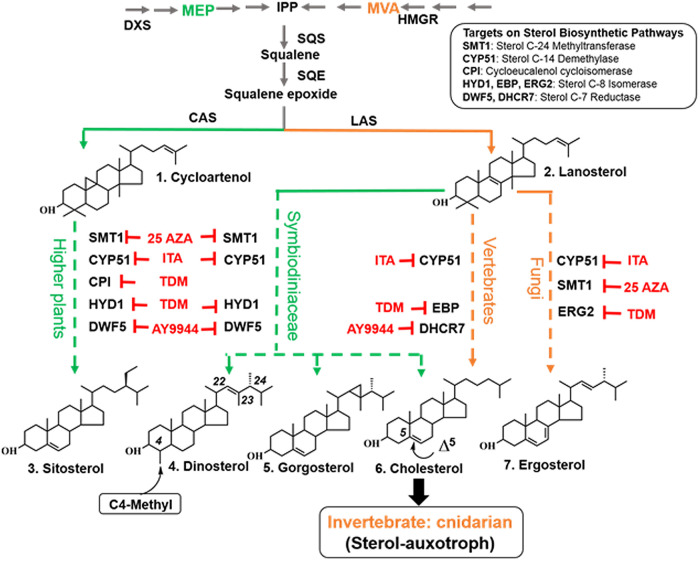
Deduced sterol biosynthesis pathways in Symbiodiniaceae. Enzyme abbreviations: DXS, 1-deoxy-d-xylulose 5-phosphate synthase; HMGR, hydroxy-methyl-glutaryl-CoA (hydroxy-methyl-glutaryl-coenzyme A) reductase; CAS, cycloartenol synthase; LAS, lanosterol synthase. Metabolite abbreviations: MEP, methylerythritol phosphate; IPP, isopentenyl pyrophosphate; MVA, mevalonic acid. Note the following. (i) In animals and fungi, the cytoplasmic MVA pathway (indicated in orange) is the only route for biosynthesis of IPP whereas IPP can be derived via either the MVA pathway or the plastidial MEP pathway (indicated in green) in higher plants. Most microalgae (including Symbiodiniaceae) have only the MEP pathway (41). (ii) Animals and fungi cyclize oxidosqualene to lanosterol by LAS as the first cyclic intermediate in sterol biosynthesis, whereas photosynthetic organisms (e.g., plants and microalgae) convert the same substrate to cycloartenol by CAS (8). (iii) LAS is found in *Arabidopsis* but has only a minor role (45). Members of the Symbiodiniaceae (e.g., B. minutum) have LAS, but not CAS. Abbreviations for inhibitors: 25 AZA, 25-azalanosterol; ITA, itraconazole; TDM, tridemorph; AY9944, a human 7-dehydrocholesterol inhibitor.

Application of well-characterized sterol biosynthetic inhibitors (with thoroughly established specificities; see Fig. 2 and Materials and Methods for the target enzymes) led to reduced levels of total sterols (Fig. S3a; see also Table 3) and a concomitant decrease in growth of B. minutum (Fig. S3b). Therefore, impairment of sterol biosynthesis seems to compromise growth of the alga. The primary target of the fungicide tridemorph (TDM) is cycloeucalenol cycloisomerase (CPI), but it also affects other isomerization steps in the pathway catalyzed by sterol C-14 isomerase (also known as HYD1 in higher plants, EBP in vertebrates, and ERG in fungi; Fig. 2). Application of this inhibitor allows us to probe for the presence of enzymes with functions similar (with or without amino acid sequence similarity) to those of CPI (37). The cycloartan structure was not observed in the sterols that accumulated in the tridemorph-treated cells (Table 3). Instead, a series of Δ^8^ sterols with C4-methylation (P-1, P-2, and P-3 [Table 3; see Fig. S2d for molecular structures]) were identified in the treated cells but in not the controls, indicating that tridemorph inhibited C8 sterol isomerase (HYD1; Fig. 2), and C4-methyl sterols generated via activity of STRM. In addition, Symbiodiniaceae genomes harbor no genes encoding CPI (Fig. S1b). These results support the hypothesis that the dinoflagellate endosymbionts lack a *CPI* gene but possess a *STRM* gene. Cross-kingdom sequence alignment revealed the presence of genes in the B. minutum genome encoding putative proteins with conserved signature amino acid motifs of lanosterol synthase (LAS), but not cycloartenol synthase (CAS) (Fig. S4). As lanosterol processing does not require *CPI*, this hypothesis is thus also supported by the *in silico* evidence (absence of *CAS*) and previous biochemical evidence (38–40), which jointly show that dinoflagellates use LAS but not CAS.

**TABLE 3:** Sterol compositions of inhibitor-treated *B. minutum*^*a*^

Sterol	Structure^*b*^	% of total sterols for indicated sterol biosynthetic inhibitors				
		Ctrl	TDM	AY9944	25 AZA	ITA
24-Nor-23-demethylgorgsterol	**B1 (H1)**	1.1	0.9	1.3	1.2	0.8
Cholesterol	**B2 (H2)**	11.4	9.6	9.5	6.1	9.2
23,24-Demethylgorgsterol	**B3**	0.6	0.7	0.5	0.5	0.5
Cholesta-5,24-dienol	**R-1**	—^*c*^	—	—	4.7	—
Brassicasterol	**B4 (H4)**	3.0	3.1	2.6	1.6	2.5
Ergosterol	**G-1**	—	—	2.6	—	—
Cholesta-8,22,24-trienol	**R-2**	—	—	—	5.4	—
Lophenol	**B5 (F2)**	0.3	1.2	1.3	0.1	0.1
4,14-Dimethylcholesta-8,22,24-trienol	**L-1**	—	—	—	—	2.4
22-Dihydrobrassicasterol	**B6**	5.7	3.7	3.1	1.8	5.1
4,14-Dimethylcholesta-8,24-dienol	**L-2**	—	—	—	—	4.2
24-Demethylgorgsterol	**B7**	2.2	2.4	1.7	1.3	2.7
4-Methylcholest-8(14),24-dienol	**R-3**	—	—	—	3.7	—
4,24-Dimethylcholest-8,22-dienol	**P-1**	—	1.7	—	—	—
Lanosta-7,9-dienol	**R-4**	—	—	—	3.0	—
24-Demethyldinosterol	**B8**	23.7	21.3	24.0	16.4	19.9
Lanost-8-enol	**R-5**	—	—	—	2.9	—
4,24-Dimethylcholest-8,24-dienol	**P-2**	—	0.7	—	—	—
4,24-Dimethylcholesta-7,22-dienol	**B9**	0.3	0.6	2.5	0.4	0.2
4,24-Dimethylcholest-7-enol	**B10 (F5)**	0.5	4.7	7.2	7.5	0.7
Obtusifoliol	**L-3**	—	—	—	—	5.2
23,24-Demethylcholesta-5,22-dienol	**B11**	8.8	10.0	8.2	15.9	6.8
4,24-Dimenthylcholestanol	**B12 (H16, F6)**	10.4	7.6	6.8	6.1	8.9
4,24-Dimethylcholest-8-enol	**P-3**	—	1.0	—	—	—
Dinosterol	**B13 (F7)**	10.7	7.6	8.8	7.4	10.6
Gorgosterol	**B14**	21.4	23.0	19.9	14.0	20.2

Total sterols (μg·mg^−1^ DW)		14.7	11.0	10.1	12.2	10.8

a Algal cells at a concentration of approximately 1 × 10^6^ cells·ml^−1^ were harvested, washed with sterile seawater, and inoculated into fresh medium with the same initial cell concentration of 2 × 10^5^ cells·ml^−1^. Cultures were acclimated for 12 h under 50 μmol photons m^−2^ s^−1^ light. Subsequently, cultures were treated with inhibitors or an equivalent amount of DMSO (not exceeding a final concentration of 0.1%; Ctrl). Cell aliquots were collected for sterol profiling four days later. Three biological replicates of algal cultures were established under each set of treatment conditions. Abbreviations: Ctrl, controls; 25 AZA, 25-azalanosterol; TDM, tridemorph; ITA, itraconazole.

b The structure numbers (in boldface) and their corresponding mass spectra are listed in Fig. S2d.

c Dashes (—) indicate values below the detection limit under the experiment conditions.

10.1128/mSystems.00765-20.3 FIG S3 Changes in sterol content (a) and growth (b) of Breviolum minutum following incubation with the indicated chemical inhibitors. Values represent means of results from three replicates. Abbreviations for inhibitors: 25 AZA, 25-azalanosterol; TDM, tridemorph; ITA, itraconazole. Asterisks (*) indicate significant differences compared with the control conditions (*P* ≤ 0.05). Download FIG S3, PDF file, 0.04 MB. Copyright © 2020 Lu et al. 2020 Lu et al. This content is distributed under the terms of the Creative Commons Attribution 4.0 International license.

10.1128/mSystems.00765-20.4 FIG S4 Sequence alignment of cycloartenol synthase (CAS) and lanosterol synthase (LAS). The conserved regions that distinguish these two proteins are shown in the red box. Bm, Breviolum minutum; At, Arabidopsis thaliana; Pj, Panax ginseng; Lj, Lotus japonicas; Sc, Saccharomyces cerevisiae; Dd, Dictyostelium discoideum; Bp, Bathycoccus prasino; Ot, Ostreococcus tauri; Cr, Chlamydomonas reinhardtii; Cs, Coccomyxa subellipsoidea C-169; Gn, Goniophlebium niponicum; Am, Abies magnifica; Av, *Avenaventricosa*; Si, Solanumlycopers icum. Note that the Fugacium kawagutii LAS candidate was omitted in the figure due to its low genome sequence quality. Download FIG S4, PDF file, 0.1 MB. Copyright © 2020 Lu et al. 2020 Lu et al. This content is distributed under the terms of the Creative Commons Attribution 4.0 International license.

Following treatment with 25-azalanosterol, which inhibits sterol methyltransferase SMT1 activity (37), levels of 24-desmethyldinosterol (B8 in Table 3; a sterol lacking the C-24 methyl group; see Fig. S2d for molecular structure) decreased from 23.7% to approximately 16.4% of the alga’s total sterol contents (Table 3). This suggests that SMT1 is responsible for alkylation of sterol side chains in B. minutum. Most of the accumulated sterols have a lanostan structure (R4 and R5 in Table 3; see Fig. S2d for molecular structures), but not cycloartan derivatives, providing additional evidence along with the bioinformatic and biochemical findings indicating that B. minutum uses LAS but not CAS (39, 40). The findings also provide indirect evidence that B. minutum has no CPI, but this inference requires validation by a cell-free enzyme assay. The 14-methyl sterols (i.e., L-1, L-2 and L-3 in Table 3; see Fig. S2d for molecular structures) accumulated in the cells following ITA application, verifying that ITA is a potent inhibitor of CYP51 (36) and can dramatically inhibit overall sterol biosynthesis (Table 3).

The compound Ay9944 inhibits the human enzyme 7-dehydrocholesterol (DHCR7) involved in cholesterol production. Members of the Symbiodiniaceae express the DHCR7 homolog DWF5, responsible for catalyzing reduction of cholesterol’s Δ^7(8)^ double bond (41). Ergosterol was detected in cells after AY9944 treatment (G1 in Table 3; see Fig. S2d for molecular structure), corroborating the similarity of the biological activity of DWF5 to that of DHCR7, as ergosterol levels generally rise after DHCR7 inhibition (42). Although some inhibitors target both “plant”-type and vertebrate/fungus-type homologues (for example, TDM affects HYD1, EBP, and ERG2; Fig. 2), the *in silico* analysis (Fig. S1b), together with evidence of increased levels of the precursor and decreased levels of downstream products caused by metabolic blocking (Table 3), allowed us to construct a putative sterol biosynthesis pathway in B. minutum (Fig. 2). In summary, the sterol biosynthetic pathway in B. minutum exhibits features both common with and distinct from the pathways in fungi, animals, green plants (including green algae and land plants), and documented microalgae (Fig. 2 legend). These findings add another layer of complexity and diversity to our knowledge of algal sterol metabolism and its role in symbiosis.

### Stress-dependent shifts in sterol biosynthesis of dinoflagellate endosymbionts in response to environmental cues.

We also probed possible effects of abiotic stresses on synthesis of sterols in dinoflagellate endosymbionts, and potential links between such effects and cnidarian bleaching. For this, we examined changes in the expression of sterol biosynthetic genes and sterol profiles in B. minutum cultures in response to three bleaching-inducing stimuli: high light, high temperature, and acidification. In these experiments, levels of gene transcripts or sterols in the stressed cells were compared with those in unstressed controls at corresponding time points.

**FIG 3:**
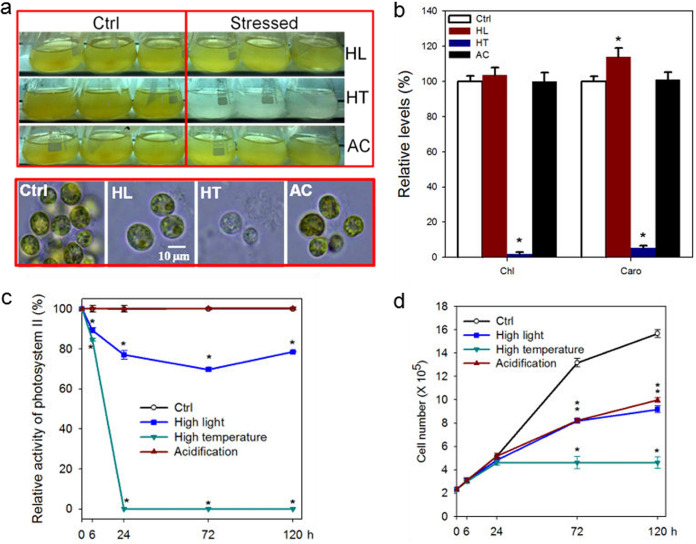
Responses of B. minutum to high-light (HL), high-temperature (HT), and acidification (AC) stresses. Algal cells at a concentration of approximately 8 × 10^5^ cells·ml^−1^ were acclimated for 12 h under 50 μmol photons m^−2^ s^−1^ light and then transferred to the designated conditions. Algal cells that were not subjected to the stresses served as controls (Ctrl). Three biological replicates of algal cultures were established under each set of treatment conditions. (a) Phenotypic changes of B. minutum in response to HL, HT, and AC stresses. (b) Pigment contents under indicated stress conditions. Chl, chlorophylls; Caro, carotenoids. (c) Changes in relative levels of maximum photosynthetic efficiency of photosystem II (PSII) of B. minutum in response to HL, HT, and AC stresses. (d) Growth curves of B. minutum in response to HL, HT, and AC stresses. Asterisks (*) indicate significant differences compared with the control conditions (*P* values ≤ 0.05).

Pigment loss, including dramatic declines in both chlorophyll and carotenoid levels, occurred in B. minutum within 24 h of exposure to high temperature, accompanied by reductions in photosynthetic efficiency and growth rate (Fig. 3a to d). In contrast, no apparent pigment loss occurred under high light within the time frames included in this study (Fig. 3b), despite observable reductions in rates of both photosynthesis (Fig. 3c) and growth (Fig. 3d). Moreover, levels of carotenoids increased (Fig. 3b), possibly as part of the adaptive suite of responses of dinoflagellate endosymbionts to high light (43). Thus, pigment bleaching is probably not the only reason for stress-related reductions in photosynthesis rates of B. minutum. In addition, the acidification stress led to a reduction in the growth rate (Fig. 3d) but did not result in either pigment bleaching (Fig. 3a and b) or a reduction in the photosynthesis rate (Fig. 3c), suggesting that reduction in photosynthesis rates was not the sole reason for the dinoflagellate’s growth arrest.

**FIG 4:**
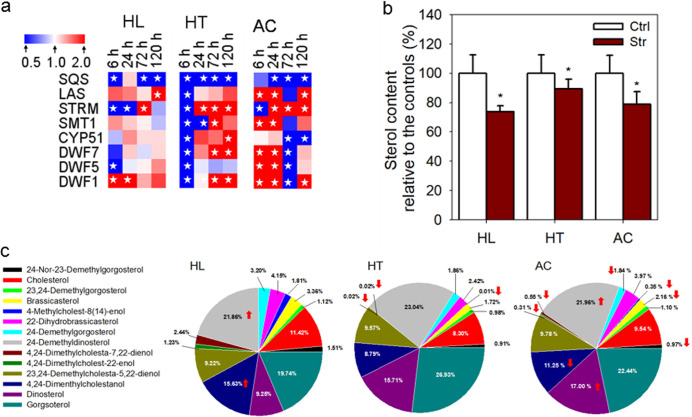
Changes in transcript levels of and sterols of B. minutum in response to high-light (HL), high-temperature (HT), and acidification (AC) stresses. For the levels of either gene transcripts or sterol profiles, the values of the stressed cells were compared with the control samples (not subjected to the stress) at the corresponding time points. Three biological replicates of algal cultures were established under each set of treatment conditions. (a) Transcriptional dynamics of sterol biosynthetic genes in response to HL, HT, and AC stresses. Red and blue indicate up- and downregulated genes, respectively. Significant differences (*P* ≤ 0.05 and fold change ≥2 or ≤0.5) in expression between stressful and nonstressful conditions at the selected time points are indicated by asterisks in the heat map. (b) Relative changes in total sterol contents induced by HL, HT, and AC stresses. Ctrl, controls; Str, stresses. Asterisks (*) indicate significant differences compared with the control conditions (*P* ≤ 0.05). (c) Changes in sterol profiles induced by HL, HT, and AC stresses. The arrows indicate increases or decreases in levels of the corresponding sterols with *P* ≤ 0.05. Please see details in Data Set S2 and note that for individual stress treatment, experiments were not conducted at the same time, which might have led to batch variations among the controls.

*SQS* transcript levels rapidly declined following the onset of each stress and remained suppressed thereafter (Fig. 4a). In contrast, following stress induced by high light and acidification, a possible feedback upregulation of the *LAS* gene in response to the decline in levels of sterol end products associated with the decrease in *SQS* activity was observed (Fig. 4a). The transcriptional levels of the genes involved in synthesis of postsqualene sterols changed in response to stress, while their expression patterns seen under conditions of different stresses markedly differed. Upon exposure to high light, *DWF1* transcripts were transiently upregulated and remained at a relatively constant level thereafter (Fig. 4a). Transcript levels of the remaining genes were relatively constant, except those of *STRM* and *DWF5*, which were rapidly downregulated within 6 h following the onset of high light (Fig. 4a). High temperature reduced levels of transcripts of all probed postsqualene biosynthetic genes within 6 h (Fig. 4a). Thereafter, transcript levels of these genes either remained constant (*DWF5*) or were increased at 120 h (*STRM*, *SMT1*, *CYP51*, *DWF7*, and *DWF1*) (Fig. 4a). Under the acidification conditions, transcripts of *STRM* were transiently downregulated but showed increased levels thereafter (Fig. 4a). We observed rapid upregulation of the other genes, followed by declines to lower or constant levels (Fig. 4a). Generally, responses of postsqualene biosynthetic genes to high temperature and acidification were faster and stronger than their responses to high light.

10.1128/mSystems.00765-20.9 DATA SET S2 Sterol dynamics in *Breviolum minutum* in response to high-light, high-temperature, and acidification stresses. Please note that for the individual stress treatments, samples were collected in different batches, which could have led to batch variations among the controls. Download Data Set S2, XLS file, 0.04 MB. Copyright © 2020 Lu et al. 2020 Lu et al. This content is distributed under the terms of the Creative Commons Attribution 4.0 International license.

Sterol profiling of B. minutum was performed in parallel with the gene expression study. All 14 sterols were identified in B. minutum grown under each of the stress conditions (Data Set S2). Assessments of the total sterol contents of the cells (Fig. 4b) and the sterol types (Fig. 4c) over a 120-h period revealed distinct patterns in their relative abundances. Despite fluctuations and wide discrepancies in expression levels of various sterol-related genes, the total sterol content decreased (Fig. 4b), with a concomitant downregulation of *SQS* transcripts (Fig. 4a). Significant changes in distributions of sterol types occurred under conditions of high temperature (Fig. S5a; see also Data Set S2) and acidification (Fig. S5b; see also Data Set S2) stresses, but not high light stress (Fig. S5c; see also Data Set S2). These findings are consistent with the considerable transcriptional alterations of genes involved in postsqualene biosynthetic pathways following the onset of high temperature and acidification stresses, and much weaker changes in expression of these genes under conditions of high light stress (Fig. 4a). Observed changes in sterol profiles included significant reductions in levels of brassicasterol (B4) under conditions of acidification or high-temperature stresses relative to the controls (Data Set S2). Hence, stress responses of B. minutum depend on the stress, and include distinct shifts in sterol biosynthesis. Thus, rates of both sterol transcription and metabolism appear to be regulated in dinoflagellate responses to environmental stresses, which are well-known causes of coral bleaching.

10.1128/mSystems.00765-20.5 FIG S5 Dynamics of sterol profiles of Breviolum minutum in response to high-temperature, acidification, or high-light stresses. The values determined for the stressed cells were compared with those determined for the control samples (not subjected to the stress) at the corresponding time points. Three biological replicates of algal cultures were established under each set of treatment conditions. Arrows indicate significant differences compared with the control conditions (*P* ≤ 0.05). Please see details in Data Set S2 and note that for the individual stress treatments, experiments were not conducted at the same time, which could have led to batch variations among the controls. (a) Dynamics of sterol profiles of B. minutum in response to high-temperature stresses. (b) Dynamics of sterol profiles of B. minutum in response to acidification stresses. (c) Dynamics of sterol profiles of B. minutum in response to high-light stresses. Download FIG S5, PDF file, 0.2 MB. Copyright © 2020 Lu et al. 2020 Lu et al. This content is distributed under the terms of the Creative Commons Attribution 4.0 International license.

## DISCUSSION

### Sterol metabolites of B. minutum, but not *F. kawagutii*, are amenable to coral hosts.

Dinostane (including dinosterol and 24-demethyldiosterol) is the predominate sterol in both B. minutum and *F. kawagutii*, and is considered to be a molecular marker for dinoflagellates (44). However, there are differences in the sterol profiles of these two strains. The sterols detected in *F. kawagutii* were exclusively C4-methylsterols. However, neither a capacity for sterol C4 methylation nor a capacity for C4 demethylation was detected in the cnidarians. Therefore, *F. kawagutii* is likely to be an inadequate source of sterols for cnidarian hosts, which require either cholesterol or other Δ^5^ sterols. These observations provide independent support for recent genomic indications that *F. kawagutii* could not match the metabolic capabilities of a coral host (5). In contrast, B. minutum produces considerable amounts of Δ^5^ sterols, including cholesterol; thus, it is better adapted for supporting growth of the cnidarian.

### Symbiodiniaceae synthesize lanosterol but not cycloartenol.

The plant sterol pathway has fundamental differences from that of metazoans. Animals and fungi cyclize oxidosqualene to lanosterol as the first cyclic intermediate in sterol biosynthesis, whereas plants, microalgae, and many protozoa convert the same substrate to cycloartenol (8). Cycloartenol is regarded as a specific marker for photosynthetic organisms. Contradicting this notion, a dual sterol synthesis pathway through lanosterol and cycloartenol has been identified in Arabidopsis thaliana. However, there, the contribution of the lanosterol pathway to production of 24-alkyl-Δ^5^ sterols was found to be marginal (45). On the other hand, a cell-free enzyme assay performed before the genomic era indicated that dinoflagellates synthesize lanosterol but not cycloartenol (38–40). In this study, we identified in Symbiodiniaceae a gene encoding LAS with conserved signature amino acid residues distinct from those in CAS, providing genomic evidence that Symbiodiniaceae enzymes have LAS but not CAS. Moreover, the absence of CPI in the Symbiodiniaceae genomes and pharmacological sterol dynamics also supports the hypothesis that lanosterol, rather than cycloartenol, is the first cyclic intermediate in sterol biosynthesis in this taxon.

### Homeostasis of sterol metabolites is essential for the mutualistic partnerships between cnidarian hosts and dinoflagellate endosymbionts.

The bleaching process, which can have catastrophic effects on marine (particularly reef) ecosystems, impairs the exchange of nutrients between cnidarian hosts and their dinoflagellate symbionts. The dependency of cnidarian hosts on their symbionts for sterols may play a crucial role, as they are essential for an animal’s growth, development, and reproduction. Accordingly, NPC sterol transporters are reportedly key molecules for functional symbiosis of these taxa (22) and dysfunction of NPC2 impairs cnidarian growth (23). In this study, we found that the alga-freed anemones contained slightly more cholesterol than the whole animals, suggesting that H. crispa might accumulate cholesterol either by selective transfer or by converting algal sterols to cholesterol via dealkylation in a manner similar to that seen with other sterol-auxotrophic animals (13). Moreover, pharmaceutical depletion of algal symbiont sterols led to declined photosynthesis of the dinoflagellate and, eventually, to anemone bleaching. On the other hand, the levels of sterol contents in both Symbiodiniaceae and the cnidarian decreased under the abiotic-stress-induced bleaching conditions. Therefore, sterol homeostasis seems to play an important role in cnidarian-dinoflagellate symbiosis. An alternative possibility is that the breakdown of symbiosis could result from impairment of photosynthesis and could therefore be an effect rather than a cause of anemone bleaching. However, this possibility can be dismissed, at least under certain circumstances, because although growth was arrested, the rate of photosynthesis was unchanged under conditions of acidification stress. During the review of this paper, an independent study on sterol transport in *Exaiptasia* anemones was published (23). It indicated that canonical NPC2 homologues are “workhorses” in sterol trafficking throughout the host but that noncanonical NPC2s play key roles in the interaction between the symbiotic partners in responses to unfavorable environmental conditions (23). These results collectively suggest that sterol transfer and homeostasis in dinoflagellate endosymbionts are crucial for the integrity of the symbiotic system and involved in the bleaching process, at least in some circumstances (e.g., bleaching induced by some environmental stresses).

### Responses of Symbiodiniaceae sterol metabolism to abiotic stresses may relate to host bleaching.

SQS appears to represent a critical point in regulation of Symbiodiniaceae (i.e., B. minutum) sterol homeostasis in response to different bleaching-inducing stresses. Under conditions of high-light, high-temperature, and acidification stresses, the growth rate of B. minutum decreased (to various degrees) and *SQS* transcripts were downregulated with a concomitant decrease in the total sterol content. These findings are consistent with the notion that a decline in sterol synthesis is associated with *SQS* activity, which is a major control point for carbon flow and sterol end product formation (46). Sterol profiles provide informative biomarkers of the integrity of cnidarian-dinoflagellate symbioses, and SQS may serve as an indicator of their functional status and the bleaching process. Sterols thus seem to be involved in the acclimatization of B. minutum to abiotic stresses, while the reduction in total sterol content seen following bleaching-inducing stresses was likely associated with the reductions in growth and overall health of dinoflagellate endosymbionts.

As genes in the postsqualene biosynthetic pathway act downstream of lanosterol, which is involved in synthesis of different sterol molecules, these genes tend to be involved in determining sterol profiles rather than total sterol contents (8). Distinct dynamics of sterol profiles were observed in responses to different environmental stresses, indicating that B. minutum has different adaptive responses to different stimuli. During thermal stress and bleaching, in the coral Acropora aspera, turnover of hormones derived from steroids in both the symbiont and host closely resembled patterns associated with brassinosteroid and glucosinolate signaling networks in terrestrial plants (47). Therefore, it could be interesting to study the roles of individual sterol derivates as signal molecules in these symbionts, where they might perform comprehensive regulatory actions affecting many biological processes, including stress responses and the breakdown of symbiosis.

Considering the evidence obtained in this study, we propose that global climate change (including increases in light intensities, temperature, and CO_2_ concentrations in seawater) could lead to disruptions in sterol exchange between cnidarian hosts and their zooxanthella endosymbionts and thus could affect maintenance of coral reef ecosystems. Future studies should further explore the causal relationship between sterol biosynthesis disruption in algal symbionts and environmentally induced bleaching of the host. Moreover, it would be interesting to elucidate more details of sterol transport and utilization in cnidarian hosts to identify novel strategies to counter cnidarian bleaching.

## MATERIALS AND METHODS

### Algal strains, growth conditions, and physiological measurements.

B. minutum strain NIES-3808 and *F. kawagutii* strain CCMP2468 were obtained from the National Institute for Environmental Studies (Japan) and the Bigelow Laboratory for Ocean Sciences (United States), respectively. They were cultured routinely in 250-ml conical flasks with 100 ml L1 medium (6) containing ampicillin (100 mg · liter^−1^), kanamycin (50 mg · liter^−1^), and streptomycin (50 mg · liter^−1^) to minimize the growth of bacteria. When culturing microalgal cells, the control group was maintained under the organism’s preferred physiological conditions (pH 8.2, 25°C, and 50 μmol·photons·m^−2^·s^−1^ light intensity) as described in earlier reports (6, 48). Under otherwise identical high-light, high-temperature and acidification stress conditions, the light intensity, temperature, and pH were set at 200 μmol·photons·m^−2^·s^−1^, 34°C, and pH 7.6, respectively, based on earlier studies (48–54) and our previous tests (48).

Algal cells at a concentration of approximately 1 × 10^6^ cells·ml^−1^ were harvested, washed with sterile seawater, and inoculated into fresh medium in triplicate. Cultures were started with the same initial cell concentration of 2 × 10^5^ cells·ml^−1^, acclimated for 12 h under 50 μmol photons m^−2^ s^−1^ light, and then exposed to designated stress conditions. Growth was monitored by measuring the cell number at 6, 24, 72, and 120 h after transfer. Cell aliquots were diluted, and cell numbers were determined under a microscope using a counting chamber (55). *F_v_*/*F_m_* values of the zooxanthellae were measured with a chlorophyll fluorometer (PAM100; Walz, Germany) as described in previous publications (55, 56). Briefly, samples were acclimated to dark for 12 min before measurement of the maximum quantum yield of photosystem II (PSII) fluorescence (*F_v_*/*F_m_* = *Fm* − *Fo*/*Fm*) with a brief saturating-light pulse (400 ms, 3,000 μmol·photons·m^−2^·s^−1^) was performed. The continuous 1.6-kHz excitation light of the fluorometer had a peak wavelength of 470 nm (intensity, <1 μmol·photons·m^−2^·s^−1^). Chlorophyll fluorescence at ≥645 nm was detected via a photodiode, and the zero offset of the fluorometer was adjusted with a filtered (0.2-μm-pore-size) medium blank each day before sampling. All fluorescence measurements were performed at room temperature (24 to 26°C). Pigments were extracted and measured as previously reported (57).

### Growth conditions and preparation of anemone samples.

H. crispa anemones were maintained in artificial 30‰ seawater (pH 8.2 to 8.3) in a recirculation aquaculture system (120 by 50 by 60 cm) under controlled conditions (24 to 26°C, 16-h/8-h light/dark cycles with approximately 50 μmol·photons·m^−2^·s^−1^ light during the light phases). Seawater was prepared with sea salt (Tropic Marin; Germany), filtered using 1-μm-pore-size filters, and renewed weekly. Healthy cultures were transferred into fresh media and acclimated for at least 2 months before experiments were performed. Specimens were fed with brine shrimp semimonthly and then starved approximately 10 days before the experiments to avoid sample contamination by food metabolites. Anemones that were not subjected to the stresses served as controls. These controls were processed in a manner identical to that used for the tested samples to maximally eliminate potential food contamination. H. crispa requires low levels of feeding for its growth and was solely dependent on the nutrients provided by endosymbionts during the experiments.

For heat treatment, the bleaching temperature was set at 34°C as described in previous reports (54, 58). As for ITA application, ITA (5 mg · liter^−1^ in dimethyl sulfoxide [DMSO]) or an equivalent amount of DMSO (not exceeding a final concentration of 0.1%) was added to the cultures. For either control or bleached organisms, tentacles were sampled from specimens using scissors and quickly dried on paper to remove excess seawater. All samples were collected at the same time of day to avoid circadian lipid shifts. Each sampled tentacle was cut lengthwise and scraped to separate the endodermal cell layer. Zooxanthella cells that live in the gastrodermis of corals were removed by scraping away the endodermic cells with a scalpel (59, 60). Suspensions were centrifuged at 300 × *g* for 3 min, and the pellets were washed with filtered seawater at least three times until all Symbiodiniaceae were released from the host cells (as confirmed by microscopic examination). Three biological replicates with four technique replicates were established for each treatment. Samples were deep-frozen in liquid nitrogen, lyophilized, weighed, and stored at −80°C until analysis.

### DNA isolation and 18S rDNA amplification.

Genomic DNA was isolated and analyzed following our previously described procedure (61). 18S rDNA fragments were amplified by PCR using two gene-specific primers, 18SF and 18SR (see Table S2 in the supplemental material) (62) and 50-μl reaction mixtures containing 1× GoTaq green master mix (Promega, USA), a 1 μM concentration of each primer, and 15 ng DNA template. Amplification conditions were as follows: an initial 95°C for 2 min, followed by 30 cycles of 94°C for 1 min, 55°C for 1 min, 72°C for 1 min·kb^−1^, and a final extension at 72°C for 5 min. The PCR products of the expected length were purified (Omega, China) and sequenced (Sangon, China).

10.1128/mSystems.00765-20.7 TABLE S2 Primers used in these experiments. Download Table S2, DOCX file, 0.01 MB. Copyright © 2020 Lu et al. 2020 Lu et al. This content is distributed under the terms of the Creative Commons Attribution 4.0 International license.

### Chemical inhibitor treatments.

Algal cultures were reinoculated with the same initial cell concentration of 2 × 10^5^, acclimated for 12 h under 50 μmol photons m^−2^ s^−1^ light, and then grown for 96 h in the presence of one of the following inhibitors targeting enzymes involved in the postsqualene sterol biosynthetic pathway: the SMT1 inhibitor 25-azalanosterol (37), CPI inhibitor tridemorph (37), CYP51 inhibitor ITA (36), and Ay9944 (which inhibits activities of human DHCR7 and Symbiodiniaceae DWF5 [63]). All chemicals were purchased from Sigma-Aldrich (Shanghai, China) with the highest purity available, unless otherwise indicated. AY9944, tridemorph, and ITA were purchased from Santa Cruz Biotechnology Co., Ltd. (Shanghai, China). The 25-azalanosterol that was used in the experiments was custom synthesized (with 90% purity) by Nanjing Sunsure Chemical Technology Co., Ltd. (Nanjing, China), following a published protocol (64).

### Analysis of transcripts related to sterol metabolism.

Algal cells at a concentration of approximately 8 × 10^5^ cells·ml^−1^ were acclimated for 12 h under 50 μmol photons m^−2^ s^−1^ light and then transferred to designated conditions. Portions of culture were collected for sterol profiling and transcript analysis 0, 6, 24, 72, and 120 h after transfer to the designated conditions. To isolate total RNA, cells were harvested by centrifugation at 6,000 rpm for 5 min, frozen in liquid nitrogen, and stored at −80°C. Total RNA was extracted using an Eastep Super total RNA extraction kit (Promega, Shanghai). The threshold cycle (2^−ΔΔ^*^CT^*) method was used to quantify relative changes in transcript levels from the quantitative PCR (qPCR) data. Levels of the transcripts under each set of treatment conditions at each time point were first normalized to actin expression levels. The values obtained for each gene were then normalized to the values in the control treatments (specimens that were not subjected to the stresses) at the corresponding time point. Values are means and standard errors obtained from three experiments. The primers used are listed in Table S2.

### Sterol extraction and identification by gas chromatography/mass spectrometry (GC/MS) analysis.

Before sterol profiling, zooxanthellae were either removed from the animals by the use of a scraping technique or not. Algal and cnidarian samples were lyophilized. Sterols were extracted and profiled as previously described (37). For sterol analysis, a 1-μl portion of each derivatized sample was injected into a GC/MS single quadrupole (GC/MSD) system. Collected data were analyzed with an Agilent GC/MSD Productivity ChemStation and AMDIS (Automated Mass spectral Deconvolution and Identification System) software. Sterols were identified by comparing their retention times and mass spectra to those of sterols from dinoflagellates and other marine organisms (65–74) and to entries in the commercial NIST/EPA/NIH Mass Spectral Library (NIST 08) GC/MS database. Sterols with similar mass spectra were identified by comparing their relative retention times with respect to cholesterol and retention indices to published values or values in the NIST database. Putative double bond positions of some sterols were determined on the basis of their fragmentation patterns and relative retention times (75–77). The mass spectra of identified sterols are listed in Fig. S3 in the supplemental material. The peak area of each sterol was integrated and its quantity in each sample was deduced by normalization with respect to the internal standard signal and dry weight of biomass. Cell pellets prepared under each set of treatment conditions at each time point were compared with the results seen with the control treatments (specimens that were not subjected to the stresses) at the corresponding time point (see Data Set S2 in the supplemental material for details). Please note that there were variations in the controls among the different treatments due to batch differences.

### Data sources and retrieval of the sterol biosynthetic enzymes.

Cnidarian and Symbiodiniaceae genome sequences were retrieved from http://reefgenomics.org. A local blast database was constructed, and inferred proteins from all analyzed genomes were subjected to blast analyses against *Arabidopsis* or human proteins. BLAST results were parsed for ≥25% amino acid identity with E values of ≤1e−10. Sequences whose real identity could not be confirmed were removed manually, and the finally selected sequences are listed in Data Set S1.

### Statistical analysis.

All experiments were replicated at least three times. Data were analyzed using analysis of variance (ANOVA) followed by paired or unpaired Student’s *t* tests. Asterisks in the figures (*) indicate *P* values of ≤0.05.
