# Supplementary figures and images for: Clade-Specific Sterol Metabolites in Dinoflagellate Endosymbionts Are Associated with Coral Bleaching in Response to Environmental Cues
Source: mSystems. 2020 Sep 29;5(5):e00765-20. doi: 10.1128/mSystems.00765-20 (PMC7527140; doi:10.1128/mSystems.00765-20)

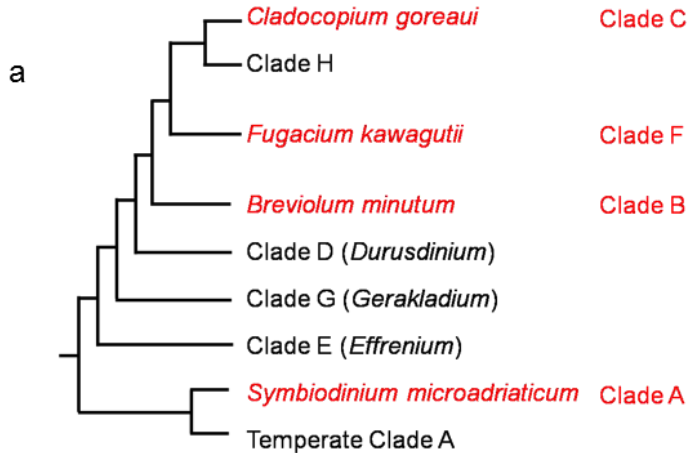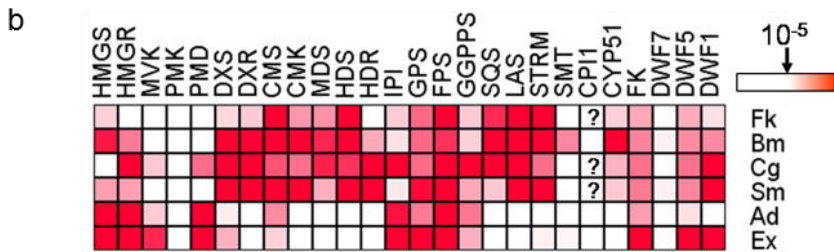

Supplement: FIG S1 [file mSystems.00765-20-sf001.pdf]

**a**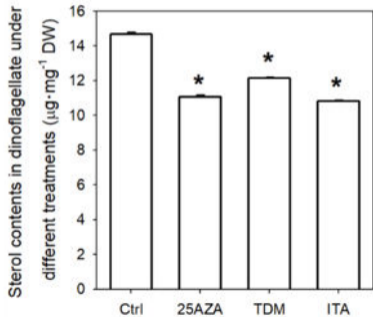**b**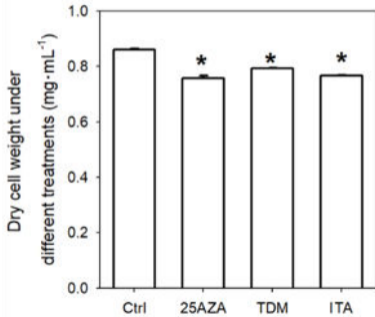

Supplement: FIG S3 [file mSystems.00765-20-sf003.pdf]

## a. High temperature

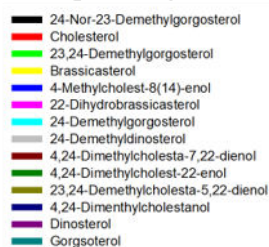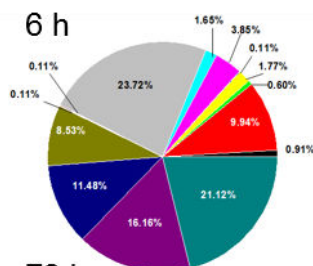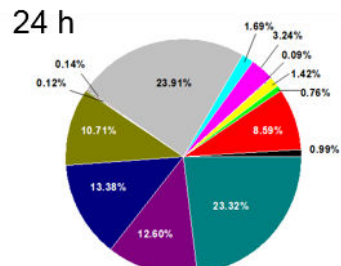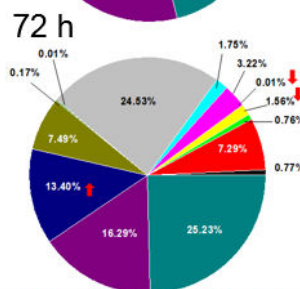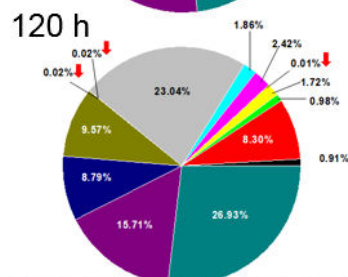

## b. Acidification

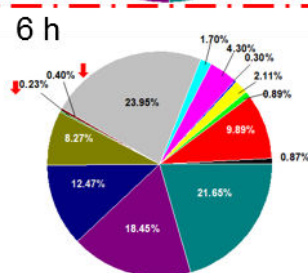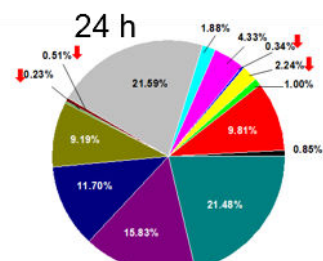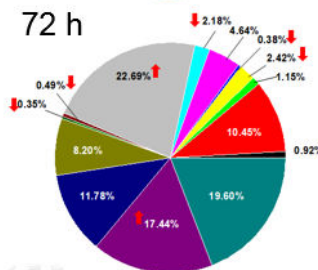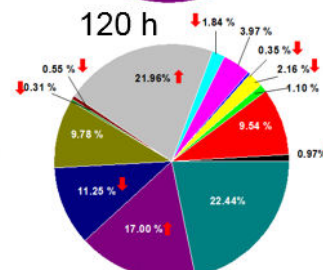

## c. High light

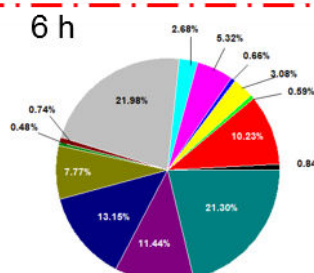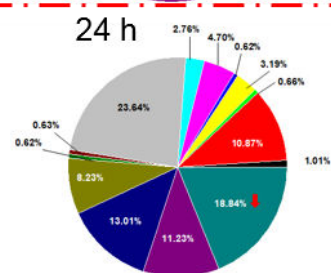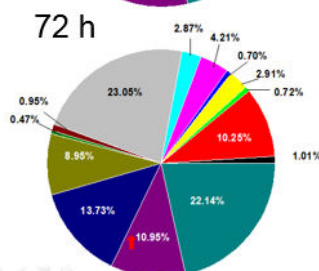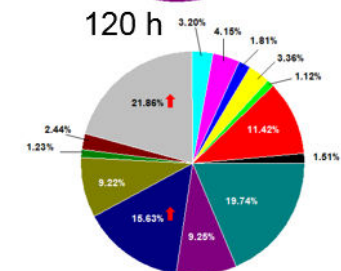

Supplement: FIG S5 [file mSystems.00765-20-sf005.pdf]
