# Supplementary material for: Clade-Specific Sterol Metabolites in Dinoflagellate Endosymbionts Are Associated with Coral Bleaching in Response to Environmental Cues
Source: mSystems. 2020 Sep 29;5(5):e00765-20. doi: 10.1128/mSystems.00765-20 (PMC7527140; doi:10.1128/mSystems.00765-20)
Supplement: FIG S2 [file mSystems.00765-20-sf002.pdf]

Figure S2.

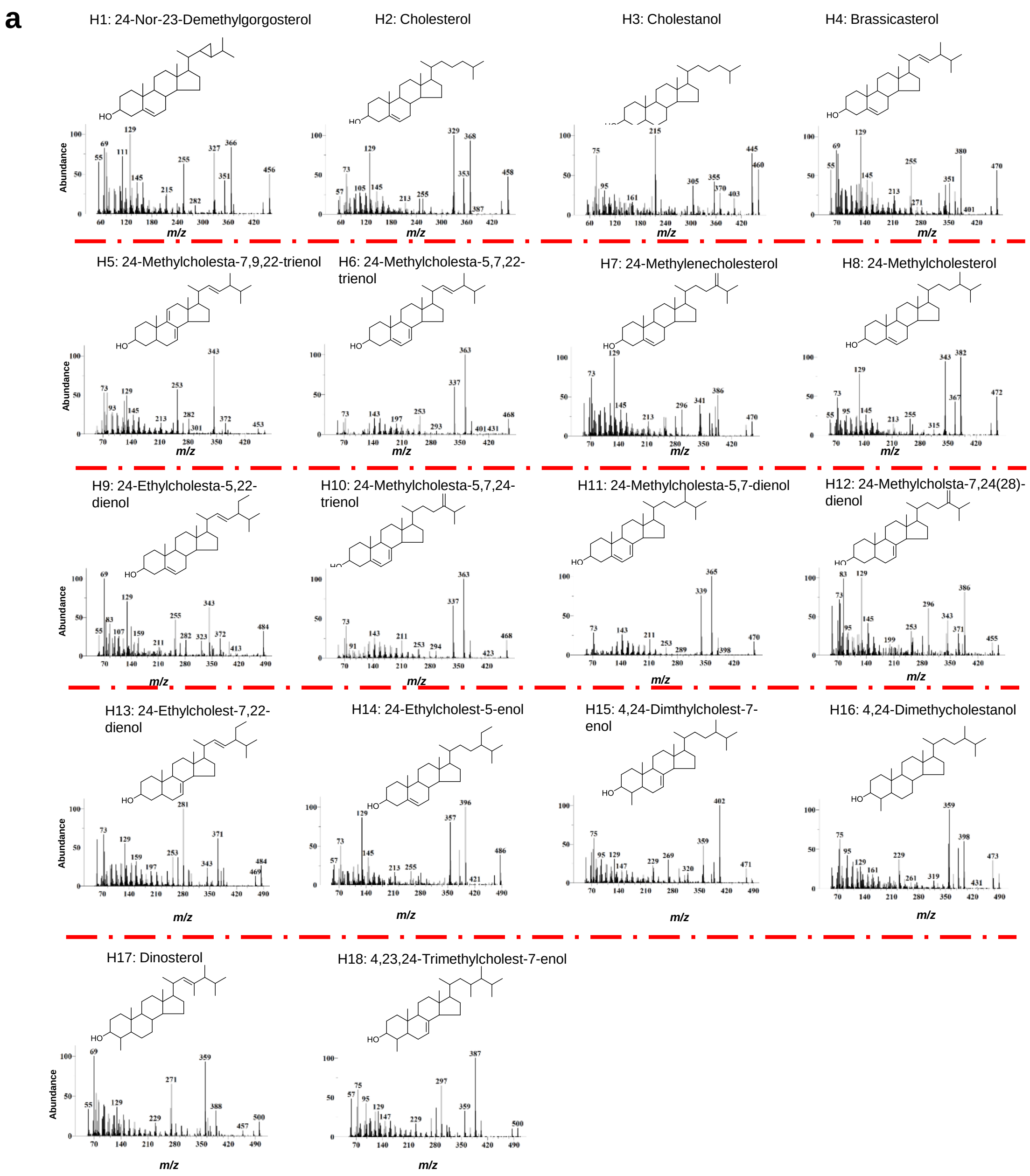
a
H1: 24-Nor-23-Demethylgorgosterol
H2: Cholesterol
H3: Cholestanol
H4: Brassicasterol
H5: 24-Methylcholesta-7,9,22-trienol
H6: 24-Methylcholesta-5,7,22-trienol
H7: 24-Methylenecholesterol
H8: 24-Methylcholesterol
H9: 24-Ethylcholesta-5,22-dienol
H10: 24-Methylcholesta-5,7,24-trienol
H11: 24-Methylcholesta-5,7-dienol
H12: 24-Methylcholsta-7,24(28)-dienol
H13: 24-Ethylcholest-7,22-dienol
H14: 24-Ethylcholest-5-enol
H15: 4,24-Dimthylcholest-7-enol
H16: 4,24-Dimethycholestanol
H17: Dinosterol
H18: 4,23,24-Trimethylcholest-7-enol
Abundance
m/z

Figure S2b

F1: 4-Methylcholest-22-enol

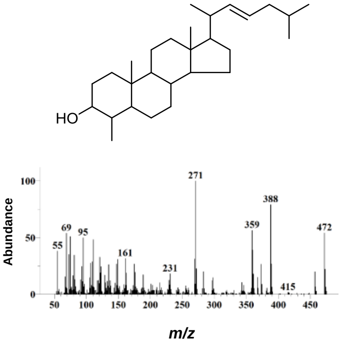

F2: Lophenol

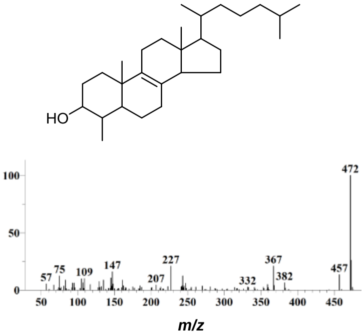

F3: Lophanol

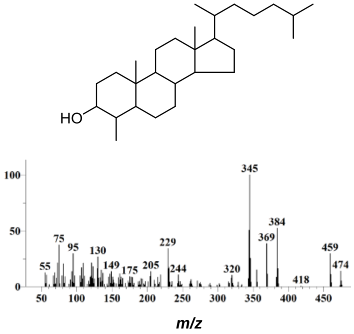

F4: 4,23-Dimethylcholest-7-enol

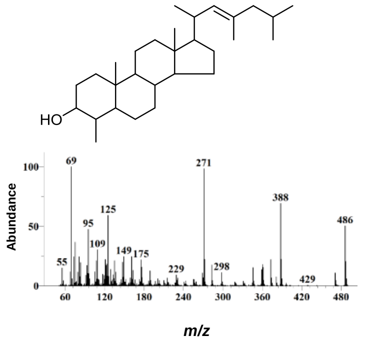

F5: 24-Demethyldinosterol

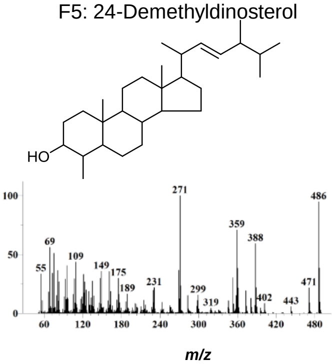

F6: 4,24-Dimenthylcholestanol

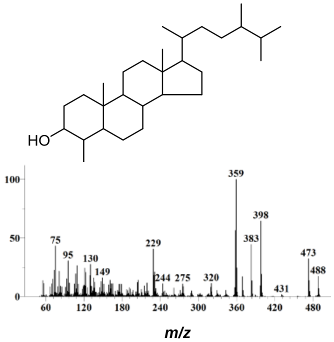

F7: Dinosterol

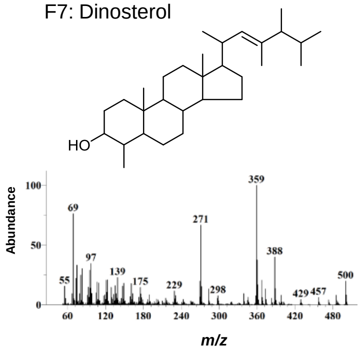

Figure S2c

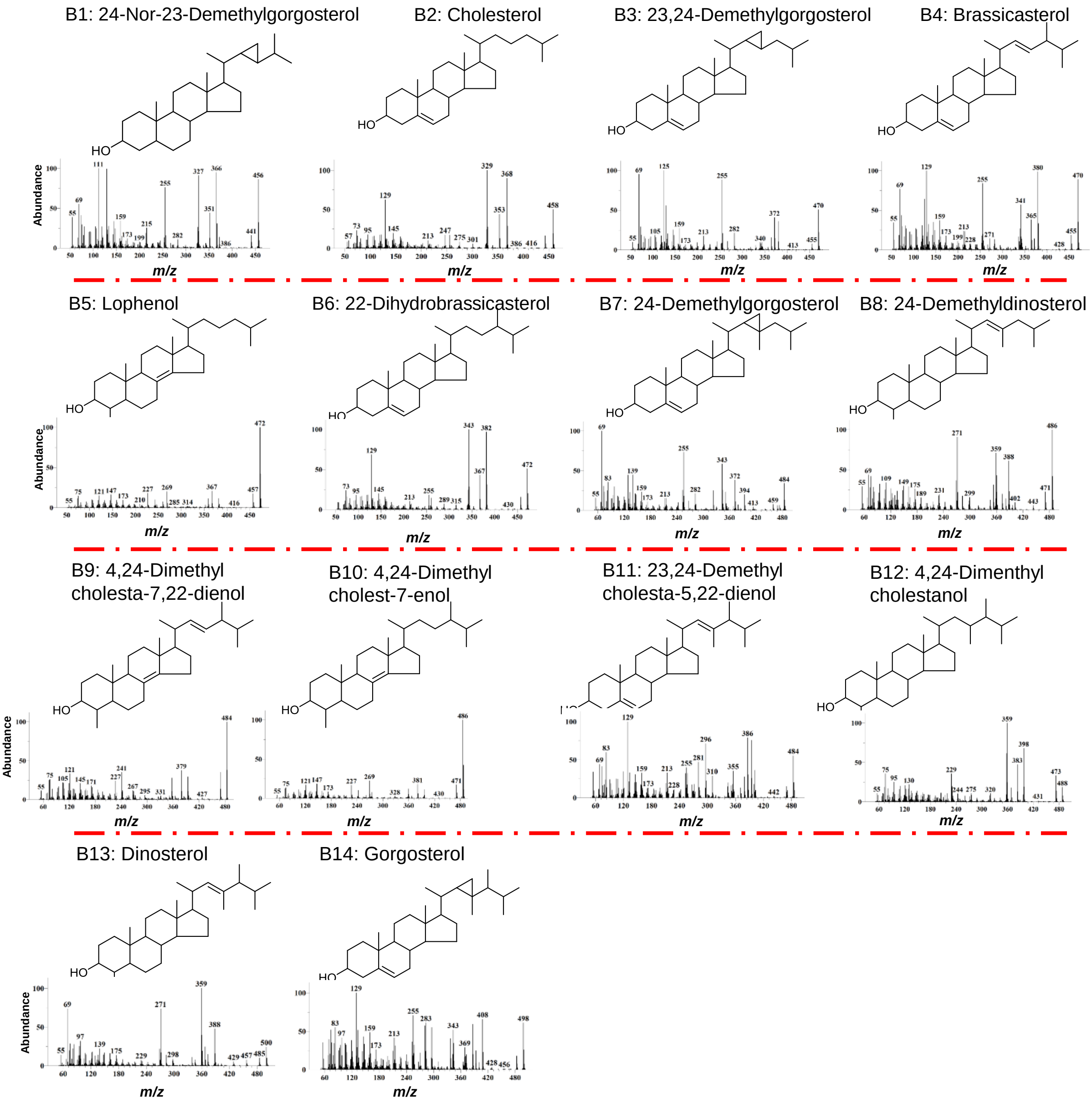
B1: 24-Nor-23-Demethylgorgosterol
B2: Cholesterol
B3: 23,24-Demethylgorgosterol
B4: Brassicasterol
B5: Lophenol
B6: 22-Dihydrobrassicasterol
B7: 24-Demethylgorgosterol
B8: 24-Demethyldinosterol
B9: 4,24-Dimethyl cholesta-7,22-dienol
B10: 4,24-Dimethyl cholest-7-enol
B11: 23,24-Demethyl cholesta-5,22-dienol
B12: 4,24-Dimenthyl cholestanol
B13: Dinosterol
B14: Gorgosterol
HO
Abundance
m/z

Figure S2d

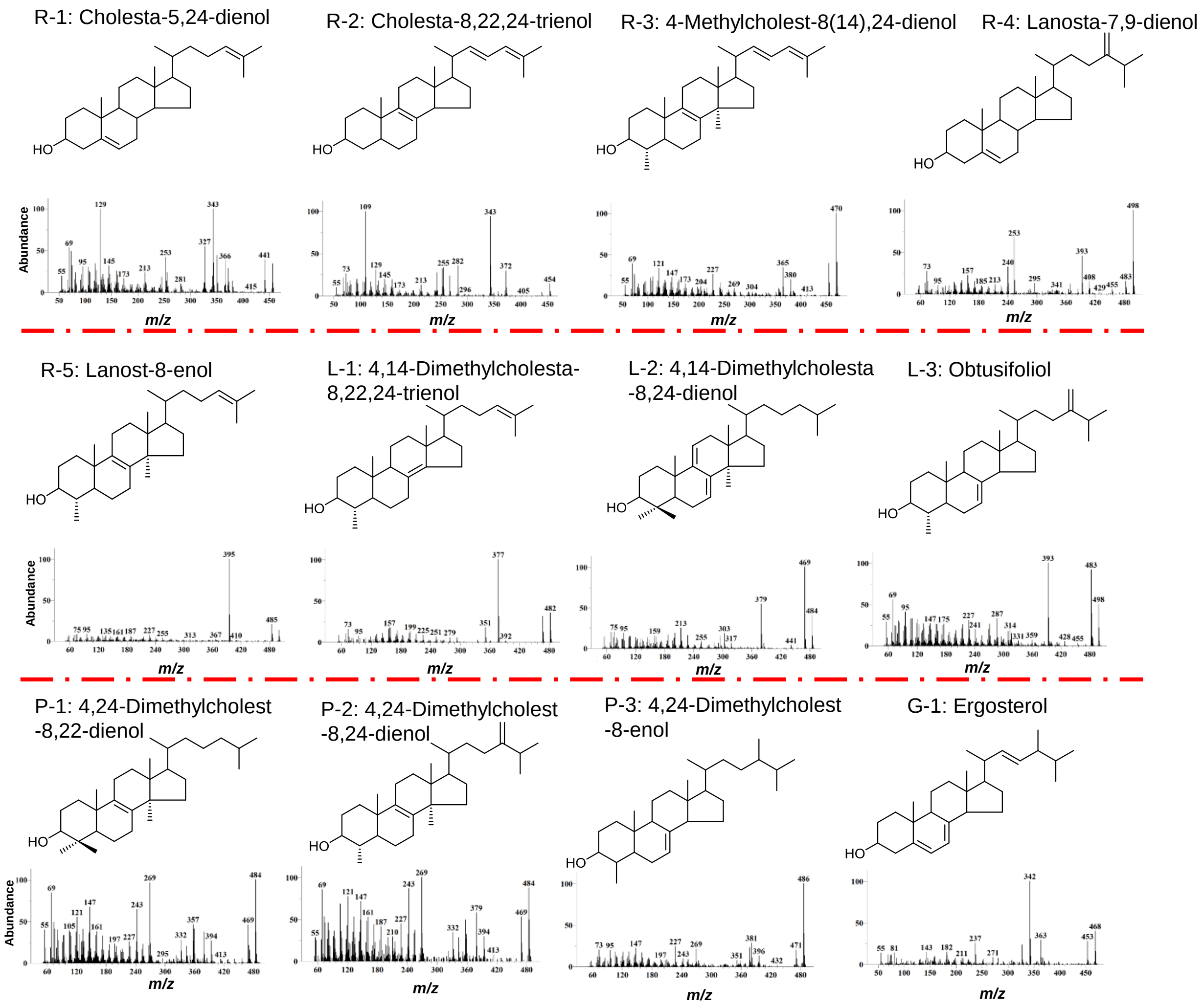

R-1: Cholesta-5,24-dienol
R-2: Cholesta-8,22,24-trienol
R-3: 4-Methylcholest-8(14),24-dienol
R-4: Lanosta-7,9-dienol
R-5: Lanost-8-enol
L-1: 4,14-Dimethylcholesta-8,22,24-trienol
L-2: 4,14-Dimethylcholesta -8,24-dienol
L-3: Obtusifoliol
P-1: 4,24-Dimethylcholest -8,22-dienol
P-2: 4,24-Dimethylcholest -8,24-dienol
P-3: 4,24-Dimethylcholest -8-enol
G-1: Ergosterol
HO
Abundance
m/z
