# Supplementary material for: Clade-Specific Sterol Metabolites in Dinoflagellate Endosymbionts Are Associated with Coral Bleaching in Response to Environmental Cues
Source: mSystems. 2020 Sep 29;5(5):e00765-20. doi: 10.1128/mSystems.00765-20 (PMC7527140; doi:10.1128/mSystems.00765-20)
Supplement: TABLE S1 [file mSystems.00765-20-st001.docx]

**Table S1**. Sterol profiles of whole or alga-freed (with symbiotic algae removed) *Heteractis crispa ^a^*

| **Sterols** | **Structures** | **Anemones (μg·mg^-1^ DW)** | |
| --- | --- | --- | --- |
|  |  | **Alga-freed** | **Whole** |
| 24-Nor-23-demethylgorgosterol | **H1(B1)** *^b^* | 0.098 ± 0.006 | 0.089 ± 0.008 |
| Cholesterol | **H2(B2)** | 1.287 ± 0.004 | 1.022 ± 0.02 |
| Cholestanol | **H3** | 0.021 ± 0.002 | 0.016 ± 0.002 |
| Brassicasterol | **H4(B4)** | 0.400 ± 0.012 | 0.280 ± 0.022 |
| 24-Methylcholesta-7,9,22-trienol | **H5** | 0.216 ± 0.004 | 0.062 ± 0.022 |
| 24-Methylcholesta-5,7,22-trienol | **H6** | 0.064 ± 0.002 | 0.060 ± 0.005 |
| 24-Methylenecholesterol | **H7** | 13.028 ± 0.110 | 11.003 ± 0.05 |
| 24-Methylcholesterol | **H8** | 1.949 ± 0.035 | 2.051 ± 0.097 |
| 24-Ethylcholesta-5,22-dienol | **H9** | 0.098 ± 0.006 | 0.065 ± 0.011 |
| 24-Methylcholesta-5,7,24-trienol | **H10** | 0.851 ± 0.006 | 0.391 ± 0.127 |
| 24-Methylcholesta-5,7-dienol | **H11** | 0.220 ± 0.002 | 0.149 ± 0.033 |
| 24-Methylcholsta-7,24(28)-dienol | **H12** | 0.017 ± 0.002 | 0.021 ± 0.008 |
| 24-Ethylcholest-7,22-dienol | **H13** | 0.428 ± 0.012 | 0.305 ± 0.013 |
| 24-Ethylcholest-5-enol | **H14** | 0.029 ± 0.002 | 0.017 ± 0.002 |
| 4,24-Dimthylcholest-7-enol | **H15(B10)** | 0.212 ± 0.010 | 0.113 ± 0.002 |
| 4,24-Dimethycholestanol | **H16(B12, F6)** | 0.108 ± 0.004 | 0.116 ± 0.000 |
| Dinosterol | **H17(B13, F7)** | 0.100 ± 0.008 | 0.049 ± 0.002 |
| 4,23,24-Trimethylcholest-7-enol | **H18** | 0.116 ± 0.010 | 0.059 ± 0.005 |
| Total sterols |  | 19.3 ±0.03 | 15.9±0.6 |

*^a^* Healthy anemones with three biological replicates were cultured in seawater with pH 8.2 at 24 - 26 ˚C and 50 μmol·photons·m^-2^·s^-1^ light. They were fed with brine shrimp semi-monthly, then starved approximately ten days before the experiments to avoid sample contamination by food metabolites. Portions of cultures were collected while zooxanthellae cells were removed by scraping technique or not for sterol profiling with three biological replicates (see ***Materials and methods*** for details). Average values with error bars are shown.

*^b^* The structure numbers in boldface and their corresponding mass spectra are listed in Miscellaneous file 1a.
