# Supplementary material for: Clade-Specific Sterol Metabolites in Dinoflagellate Endosymbionts Are Associated with Coral Bleaching in Response to Environmental Cues
Source: mSystems. 2020 Sep 29;5(5):e00765-20. doi: 10.1128/mSystems.00765-20 (PMC7527140; doi:10.1128/mSystems.00765-20)
Supplement: FIG S4 [file mSystems.00765-20-sf004.pdf]

Bm-LAS	494	EFKDMSKKAWHWLVKEQVRSLPEGDWKHWRQAIQGG-WGFSTAEQAWPVS	542
At-LAS	433	DYGLMLKKAHNYIKNTQIRKDTSGDPGLWYRHPCKGGWGFSTGDNPWPVS	482
Pj-LAS	446	EYGSMLKKAHDFIKISQVREDSPGNLSSWNRHISKGGWPFSTPDNGWPVS	495
Lj-LAS	434	EYGSMLKRANEFIKCSQITTNSSSNPSAWYRHISKGSWGFSTPDNGWPVS	483
Sc-LAS	409	EFYNTIVSAYKFLCHAQFDTECV---PGSYRDKRKGAWGFSTKTQGYTVA	455
At-CAS	433	EYGPVLEKAHSFVKNSQVLEDCPGDLNYWYRHISKGAWPFSTADHGWPIS	482
Pj-CAS	433	EYGPTLRKAHTFMKNSQVLDDCPGDLDAWYRHVSKGAWPFSTADHGWPIS	482
Lj-CAS	433	EYGPTLRKAHTFIKNSQVLEDCPGDLNKWYRHISKGAWPFSTADHGWPIS	482
Dd-CAS	389	QFQDCMKLAGHYLDISQVPEDAR-DMKHYHRHYSKGAWPFSTVDHGWPIS	437
Bp-CAS	436	NFNECLRDAAKYIDDSQVRDDAP-ELKKYYRHISKGAWPFSTRDHGWPIS	484
Ot-CAS	496	EYSDCLRLAHDYIDKSQVRDDCP-DVKKWYRHISKGAWPFSTRDHGWPIS	544
Cr-CAS	432	VSGHCLRKAHEYVEQSQVIEEAAAPLSAYYRHISKGAWPFSSRDHGWPIS	481
Cs-CAS	430	EFSDCMRRAHHYVDVTQVRDDCPGPLSAWYRHISKGAWPFSTRDHGWPIS	479
Gn-CAS	436	TCGPMLKKAHAFIDKSQVRDDCPGDLQFWYRHISKGAWPFSTRDHGWPIS	485
Am-CAS	433	DCGPLLKKAHIYIERSQVQEDCPGDLNFWYRHISNGAWPFSTRDHGWPIS	482
Av-CAS	434	EFAPTLKLAHNFIKNSQVLDDCPGDLSYWYRHISKGAWPFSTADHGWPIS	483
Si-CAS	433	EYGSTLRKAHSFLTNTQVLDNCPGDLDFWYRHISKGAWPFSTADHGWPIS	482
