# Supplementary material for: Clade-Specific Sterol Metabolites in Dinoflagellate Endosymbionts Are Associated with Coral Bleaching in Response to Environmental Cues
Source: mSystems. 2020 Sep 29;5(5):e00765-20. doi: 10.1128/mSystems.00765-20 (PMC7527140; doi:10.1128/mSystems.00765-20)
Supplement: TABLE S2 [file mSystems.00765-20-st002.docx]

**Table S2**. Primers used in this study.

| **Primer** | **Oligonucleotide Sequence 5’-3’** | **Product Size (bp)** |
| --- | --- | --- |
| 18SF | GGTTGATCCTGCCAGTAGTCATATGCTT |  |
| 18SR | AGCACTGCGTCACTCCGAATAATTCACCGG | 1700 |
| ACTIN-014253-F | CAACGGAAGTGGAATGTGC | 307 |
| ACTIN-014253-R | CTTTGGGTTCAAGGGTGC |  |
| SQS-017867-F | GACATGGACGCATACAAAGG | 259 |
| SQS-017867-R | TTCCCTGTGCCAAATCG |  |
| LAS-027161-F | GTGGCTTTGAGACTCGTGG | 174 |
| LAS-027161-R | CCACATCTCAGGAGGCACA |  |
| CYP51-021020-F | GGGGAGCGAAGTATTGTGG | 284 |
| CYP51-021020-R | ATTCCGCCGAAGAAAGC |  |
| DWF7-014997-F | CGCATTGCTGAGCGGTAT | 263 |
| DWF7-014997-R | TTGAAGCGTCTTTCGTCCT |  |
| DWF5-000104-F | AGGCACCGTTGACAAATACA | 206 |
| DWF5-000104-R | AAGGGAAAGGAGACTGTGGA |  |
| DWF1-014846-F | CCACCATCCTCATTCAGACA | 207 |
| DWF1-014846-R | CGGTCATAGGCTTCCAAACT |  |
| STRM-020967-F | AGACCAATCCCGAGCACA | 256 |
| STRM-020967-R | GGAGGAAGCAATCGCAGT |  |
| SMT1 -040208-F | GTTGCGACCGACTTCTATCTC | 229 |
| SMT1-040208-R | CGTTGATTGTGACGCCAGT |  |
